# Single extreme low dose/low dose rate irradiation causes alteration in lifespan and genome instability in primary human cells

**DOI:** 10.1038/sj.bjc.6603775

**Published:** 2007-05-08

**Authors:** M Okada, A Okabe, Y Uchihori, H Kitamura, E Sekine, S Ebisawa, M Suzuki, R Okayasu

**Affiliations:** 1 Molecular Probe Group, National Institute of Radiological Sciences, 4-9-1 Anagawa, Inage-ku, Chiba-shi 263-8555, Japan; 2 Heavy-Ion Radiobiology Research Group, National Institute of Radiological Sciences, 4-9-1 Anagawa, Inage-ku, Chiba-shi 263-8555, Japan; 3 Department of Physics, International Christian University, Mitaka-shi, Tokyo, Japan; 4 Department of Technical Support and Development, National Institute of Radiological Sciences, 4-9-1 Anagawa, Inage-ku, Chiba-shi 263-8555, Japan

**Keywords:** low dose, low dose rate, senescence, DNA double strand break, high LET radiation

## Abstract

To investigate the long-term biological effect of extreme low dose ionising radiation, we irradiated normal human fibroblasts (HFLIII) with carbon ions (290 MeV u^−1^, 70 keV *μ*m^−1^) and *γ*-rays at 1 mGy (total dose) once at a low dose rate (1 mGy 6–8 h^−1^), and observed the cell growth kinetics up to 5 months by continuous culturing. The growth of carbon-irradiated cells started to slow down considerably sooner than that of non-irradiated cells before reaching senescence. In contrast, cells irradiated with *γ*-rays under similar conditions did not show significant deviation from the non-irradiated cells. A DNA double strand break (DSB) marker, *γ*-H2AX foci, and a DSB repair marker, phosphorylated DNA-PKcs foci, increased in number when non-irradiated cells reached several passages before senescence. A single low dose/low dose rate carbon ion exposure further raised the numbers of these markers. Furthermore, the numbers of foci for these two markers were significantly reduced after the cells became fully senescent. Our results indicate that high linear energy transfer (LET) radiation (carbon ions) causes different effects than low LET radiation (*γ*-rays) even at very low doses and that a single low dose of heavy ion irradiation can affect the stability of the genome many generations after irradiation.

Ever since the seminal discovery of cellular senescence of human cells by Hayflick and Moorhead (1961), their finding has contributed substantially to understanding the mechanism of aging (Shay and Wright, 2000). Some of the molecular regulators associated with senescence have been identified (Herbig *et al*, 2004) these molecules include p21 and p16. Many years before Heyflick's finding, Henshaw *et al* (1947) showed that exposure to ionising radiation (IR) accelerated the process of aging in animal experiments. Along with other evidence, genome stability has been regarded as an important factor in the aging process (Vijg and Suh, 2006). However, premature aging by IR was not clearly demonstrated in the cell culture model demonstrated by Hayflick (Holliday, 1991). In contrast, one fairly recent study indicated the extension of lifespan of human embryo cells in culture with repeated daily doses of low level *γ*-rays, although these irradiated cells had a higher number of chromosomal genome instability than non-irradiated control (Suzuki *et al*, 1998). Recently, Suzuki *et al* (2005) demonstrated a reduction in the lifespan of normal human fibroblasts exposed to chronic low doses of heavy ion particles, whereas no reduction in lifespan under similar dose/dose rate of *γ*-rays was observed. The dose rate they used was about the level astronauts would receive during their space travel.

To further clarify this important subject of *in vitro* senescence phenomenon with IR at low doses, we exposed normal human fibroblasts to a single dose of low dose/low dose rate high linear energy transfer (LET) heavy ion irradiation and observed the cultured cells up to 5 months. Our results indicate a clear reduction in the cell's lifespan after a single dose of carbon ion irradiation, while no reduction in lifespan was observed in *γ*-irradiated cells under similar conditions. The markers of DNA double strand breaks (DSBs) were also examined in these cells as a recent study indicated the accumulation of these markers in senescent cells (Sedelnikova *et al*, 2004).

## MATERIALS AND METHODS

### Cell culture

Normal human lung fibroblasts (HFLIII) were obtained from RIKEN Cell Bank. Cells were grown in F-12 Nutrient Mixture (Ham's F-12) Medium containing 1% antibiotic–antimycotic supplemented with 15% fetal bovine serum.

### Irradiation

Cells were inoculated into a 25 cm^2^ flask and cultured until at a confluent state. Medium was changed and the flasks were filled with new medium before irradiation. Low dose (1 mGy) and low dose rate (1 mGy 6–8 h^−1^) carbon ion (290 MeV u^−1^ original energy, 70 keV *μ*m^−1^) irradiation was performed at Heavy Ion Medical Accelerator in Chiba (HIMAC) biology facility at National Institute of Radiological Sciences (NIRS). *γ*-ray irradiation was performed at a similar dose and dose rate with ^137^Cs *γ*-rays (1 mGy 6 h^−1^). As a control, non-irradiated cells were placed in HIMAC biology control room under the same conditions.

### Cell growth kinetics and immunofluorescence measurements

Cell growth kinetics was obtained by counting the number of subcultured cells using a haemocytometer at regular intervals (about 7 days) up to 5 months. Two hours after irradiation, cells were trypsinised, counted, and then reinoculated on coverslips for immunostaining. The cells on coverslips were immunostained as described previously (Okayasu *et al*, 2006). We used anti-phosphorylated DNA-PKcs (Thr 2609) polyclonal antibody (Sigma Genosys, Ishikari-shi, Japan) and an anti-*γ* H2AX polyclonal antibody (Upstate, NY, USA) as primary antibodies. As secondary antibodies, we used Cy2-conjugated AffiniPure goat anti-rabbit IgG (Jackson ImmunoResearch, West Grove, PA, USA) for DNA-PKcs, and Cy3-conjugated AffiniPure donkey anti-mouse IgG for *γ*-H2AX (Jackson ImmunoResearch).

## RESULTS

### Cell growth kinetics shows early senescence in low dose carbon-irradiated cells

**Figure 1:**
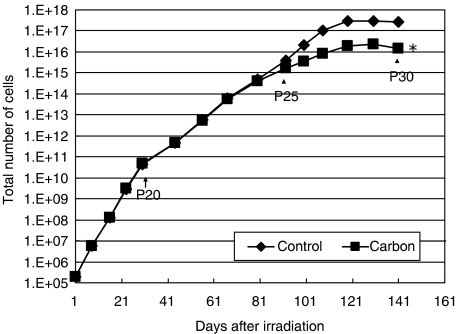
HFLIII cells were irradiated with 1 mGy carbon ions (290 MeV u^−1^ original energy, 70 keV *μ*m^−1^) at 1 mGy 6 h^−1^ and the cell growth was compared with that of non-irradiated control cells. The numbers in the figure indicate cell passage numbers. Carbon ion irradiation induced accelerated senescence at passage number around 25. (^*^P<0.05 compared to non-irradiated control cells by Student's *t*-test)

**Figure 2:**
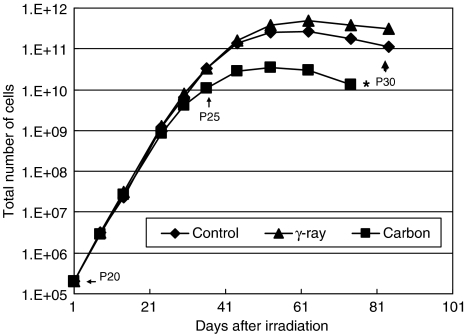
HFLIII cells were irradiated with 1 mGy carbon ions (290 MeV u^−1^, 70 keV *μ*m^−1^) at 1 mGy 7.3 h^−1^ and with 1 mGy *γ*-rays at 1 mGy 6 h^−1^, and the cell growth was compared with that of non-irradiated control cells. The numbers in the figure indicate cell passage numbers. The cells irradiated with carbon ions senesced earlier than the non-irradiated control cells, while the cells with *γ*-irradiation showed delayed senescence when compared to control. (^*^P<0.05 compared to non-irradiated control cells.) However, cells irradiated with *γ*-rays were not statistically significant (*P*=0.16) when compared with non-irradiated control.

We irradiated normal human fibroblasts with carbon ions once at 1 mGy at low dose rate (1 mGy 6 h^−1^: 0.0028 mGy min^−1^), observed the cell growth kinetics for a period of 5 months, and compared the results with non-irradiated control cells. The dose rate we used was similar to the level astronauts would be exposed to in space. The growth of irradiated cells with carbon ions started to slow down much earlier than that of non-irradiated control cells reaching senescence (Figure 1). To make certain that this is a reproducible phenomenon, we repeated the same growth experiment with cells irradiated with carbon at a similar dose and dose rate. As can be seen in Figure 2, early senescence was again observed in the carbon-irradiated cells and the slowing down of cell growth started to occur around the same cell passage number (about passage 24) as in the first experiment. The data analyses indicate that the two carbon growth curves are statistically significant when compared with non-irradiated control cell growth (see figure legend). We also examined the growth of cells irradiated with low LET *γ*-rays at a similar dose and dose rate (Figure 2). Of interest, there was no growth disadvantage in cells irradiated with *γ*-rays, and these cells showed a rather slight delay in the onset of senescence; however, this delay was not statistically significant (see figure legend). We have repeated the *γ*-ray experiment and basically obtained the same result (data not shown).

### The number of foci for DNA DSB markers starts to increase as cells reach senescence

**Figure 3:**
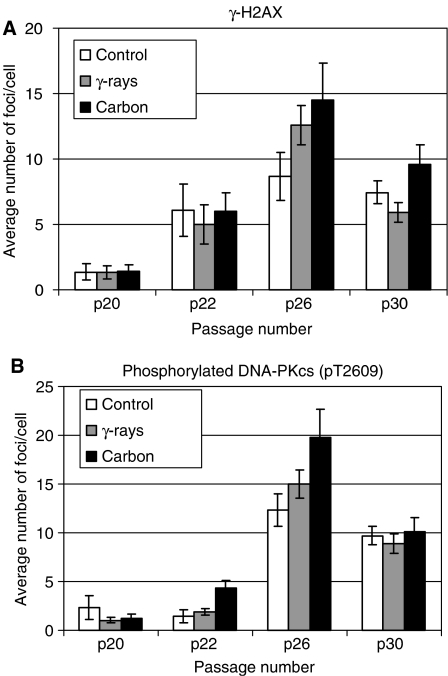
(**A**) Yield of average numbers of *γ*-H2AX foci per cell as a function of cell passage number after irradiation. The number of foci at presenescence stage (p26) was increased in all the cells and further increased especially in carbon-irradiated cells. In the fully senescent (p30) cells, the number of foci was significantly reduced. (**B**) Yield of average numbers of phosphorylated DNA-PKcs foci per cell as a function of cell passage number after irradiation. Similar tendencies as in (**A**) can be observed (*P*=0.054 between carbon and control foci numbers).

**Figure 4:**
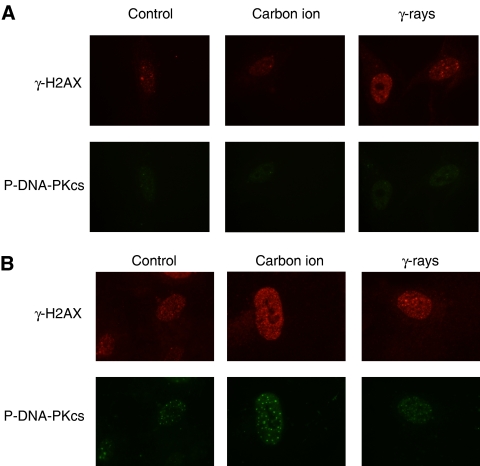
(**A**) Representative images of *γ*-H2AX foci and phosphorylated DNA-PKcs (Thr 2609) foci in cells irradiated with carbon ions and *γ*-rays along with non-irradiated control cells at passage 22. The red and green dots indicate the *γ*-H2AX foci and phospholyrated DNA-PKcs foci, respectively. (**B**) Representative images of *γ*-H2AX foci and phosphorylated DNA-PKcs (Thr 2609) foci in cells irradiated with carbon ions and *γ*-rays along with non-irradiated control cells at passage 26. The red and green dots indicate the *γ*-H2AX foci and phosphorylated DNA-PKcs foci, respectively.

Figure 3A shows yield of average numbers of *γ-*H2AX foci per cell as a function of cell passage number after cells exposure to *γ*-rays and carbon ions along with non-irradiated control. As *γ*-H2AX foci are known to correspond to DNA DSBs (Paull *et al*, 2000; Rothkamm and Lobrich, 2003), the senescence process itself seemed to produce DSBs as the number of foci increased for all the samples at passage 22, and this phenomenon was further enhanced by IR at later passages (see passage 26), especially high LET carbon ions. In order to confirm the existence of DSBs in senescing cells, we used a phospho-specific antibody for DNA-PKcs (Thr 2609) to detect an active NHEJ-type DSB repair process (Dibiase *et al*, 2000; Chan *et al*, 2002) (Figure 3B). The number of phosphorylation sites for DNA-PKcs started to increase in cells with carbon irradiation at passage 22, and although the number was further increased for all the samples, it significantly increased with carbon-irradiated samples (*P*<0.1 between control and carbon data at passage 26). Although these DSB markers could be a sensitive indicator for senescence as recently reported (Sedelnikova *et al*, 2004), it appears that DNA-PKcs phosphorylation is a better marker for senescence. The representative foci images for *γ*-H2AX and DNA-PKcs are given in Figure 4A (passage 22) and Figure 4B (passage 26). However, once cells reached the full senescence stage, the numbers of these markers were significantly reduced.

## DISCUSSION

In this report, we have shown for the first time that a single low dose/low dose rate heavy ion irradiation causes early senescence. The dose and dose rate level we used (1 mGy 6–8 h^−1^) was similar to the level astronauts would receive per day in their space exploration (about 1 mGy day^−1^). The general public could receive this dose level (1 mGy or higher), although it is not heavy ions, in a diagnostic radiology examination. In the past, a similar life-shortening phenomenon in normal human fibroblasts was reported by Suzuki *et al* (2005) after many days of chronic low dose/low dose rate charged particles; however, the accumulated dose was 200–300 mGy in their case. Thus, our finding with a single 1 mGy heavy ion exposure is unique and unexpected. We also found that a single *γ*-ray exposure at the similar dose and dose rate did not cause life shortening, but rather led to a slight extension of their lifespan. A similar tendency was reported with chronic low dose/low dose rate *γ*-ray exposure studies (Suzuki *et al*, 1998, 2005). Our senescence data with heavy ion irradiation are consistent with the animal data with neutron irradiation found by Henshaw *et al* (1947) many years ago. This would make sense as neutron irradiation has been shown to have similar biological effectiveness as heavy ions (Hall, 1982). Henshaw *et al* also showed data with *γ*-irradiation, but the life shortening was much less distinct. Our theoretical calculations indicate that in cells irradiated with carbon ions at 1 mGy 6 h^−1^, only one in eighteen cells would be hit. These data seem to indicate that the accelerated senescence caused by low dose carbon irradiation was a result of bystander effect. Bystander effects are the nontargeted effects observed in cells that were not directly irradiated, but were either in contact with or received soluble signals from irradiated cells via gap junctions. Although the effect of our carbon ion irradiation was mainly caused by the bystander effect, early senescence was clearly observed when compared to the non-irradiated control and *γ*-irradiated cells.

Sedelnikova *et al* (2004) showed that *γ*-H2AX foci accumulated in senescing human cells and in aging mice, and these foci colocalised with DSB repair proteins such as 53bp1, Mre11, Rad50, and Nbs1. They indicated that cells accumulated persistent DNA lesions that contain unrepairable DSBs during senescence. Zhang *et al* (2005) also showed histone H2A variant, macroH2A foci increased exponentially as the cells approached senescence. We confirmed their finding with the *γ*-H2AX assay and further indicated that low dose heavy ion irradiation created extra unrepaired DSBs after many days of culturing; this should not be caused by the direct hit of radiation as the sample from an earlier passage (passage 20 for example) did not show the increase. To confirm the appearance of DSB damage many days after irradiation, we used an antibody to detect the phosphorylation of DNA-PKcs, an NHEJ protein, which indicates the actual occurrence of DSB repair process. Our data clearly revealed the passage- and irradiation-dependent appearance of this phosphorylation signal, suggesting that aged cells sustained DSB, and low dose heavy ion irradiation further induced novice DSBs in late passages. We also analysed senescence-associated *β*-galactosidase, but not much difference among *γ*-ray irradiated, carbon ion irradiated, and non-irradiated control cells was observed. As mentioned before, *γ*-H2AX and DNA-PKcs foci could be more useful indicators than the senescence-associated *β*-galactosidase analysis for cell senescence. This would be the first time that the phospho-specific DNA-PKcs marker was introduced as an indicator for cell senescence. If DSBs were associated with cell senescence, the senescent status on NHEJ-deficient cells would be affected. In this regard, our preliminary results with NHEJ-deficient human fibroblasts 180BR showed further accelerated senescence than normal cells (data not shown). These results are consistent with our DSB marker results. In addition, once cells reach the full senescence stage, the signals for the DSB markers decreased significantly, indicating that the fully senesced cells have different metabolic functions (less need for repair function). A similar finding was recently reported by Bakkenist *et al* (2004). They indicated that although ATM activation and *γ*-H2AX foci formation were induced by telomere dysfunction as a stress response in late-passage presenescent cells but not in early-passage cells, they disappeared once cells become fully senescent. They concluded that fully senescent cells do not require these stress responses induced by telomere dysfunction for the maintenance of senescence. Moreover, there are a number of studies discussed about the correlation between telomere shortening (cellular senescence) and DNA damage response (d'Adda di Fagagna *et al*, 2003; Takai *et al*, 2003; Shay and Wright, 2004; von Zglinicki *et al*, 2005; Herbig and Sedivy, 2006). We showed an increase in the number of foci related to DSBs at the presenescence stage.

In summary, we showed that a single low dose/low dose rate irradiation (1 mGy 6–8 h^−1^) with heavy ion particles induced early senescence in normal human fibroblasts, while *γ*-irradiation under a similar dose/dose rate condition did not cause life shortening. DNA DSB and DSB repair markers were increased at the presenescence stage and were further enhanced in number for cells irradiated once with low doses of carbon ions. However, these DSB markers were significantly reduced once cells became fully senescent, suggesting less necessity for DNA damage/repair function in that stage.
